# Single-Access Laparoscopic Surgery for Ileal Disease

**DOI:** 10.1155/2012/697142

**Published:** 2012-03-05

**Authors:** Mohamed Moftah, John Burke, Aaditya Narendra, Ronan A. Cahill

**Affiliations:** Department of Colorectal Surgery, Beaumont Hospital, Beaumont Road, Dublin 9, Ireland

## Abstract

*Aim*. Single-access laparoscopic surgery (SALS) can be effective for benign and malignant diseases of the ileum in both the elective and urgent setting. *Methods*. Ten consecutive, nonselected patients with ileal disease requiring surgery over a twelve month period were included. All had a preoperative abdominopelvic computerized tomogram. Peritoneal access was achieved via a single transumbilical incision and a “surgical glove port” utilized as our preferred access device. With the pneumoperitoneum established, the relevant ileal loop was located using standard rigid instruments. For ileal resection, anastomosis, or enterotomy, the site of pathology was delivered and addressed extracorporeally. *Result*. The median (range) age of the patients was 42.5 (22–78) years, and the median body mass index was 22 (20.2–28) kg/m^2^. Procedures included tru-cut biopsy of an ileal mesenteric mass, loop ileostomy and ileotomy for impacted gallstone extraction as well as ileal (*n* = 3) and ileocaecal resection (*n* = 4). Mean (range) incision length was 2.5 (2–5) cm. All convalescences were uncomplicated. *Conclusions*. These preliminary results show that SALS is an efficient and safe modality for the surgical management of ileal disease with all the advantages of minimal access surgery and without requiring a significant increase in theatre resource or cost or incurring extra patient morbidity.

## 1. Introduction

There has been a recent shift in the paradigm of operative access toward minimally invasive approaches for the majority of surgical specialities. This has occurred due to the proven benefits of faster recovery times, reduced hospital stay, less wound-related complications, and better cosmesis. The recent development of single access laparoscopic surgery (SALS) represents a natural evolution in progressive practices in order to further improve patient outcomes by minimising operative wounding and reducing access-related complications and the number of ports used.

Many elective general and specialized operations for both benign and malignant diseases have now been performed using SALS techniques. The evidence from the literature to date shows it is a safe and efficient approach that, in the case of malignancy, provides adequate oncologic resection [1–3]. SALS has also been advocated as an important step in promoting safe live donor organ harvest [2, 4].

Nonetheless, compared to standard laparoscopic surgery, this approach necessitates crowding of instruments within one single incision which results in loss of triangulation. This makes the procedure challenging even for the experienced laparoscopic surgeon especially early in a department's learning curve. Moreover, the longer distance from insertion to operative site and lack of manoeuvrability present additional challenges. These challenges have discouraged many surgeons from adopting this technique [5]. This prejudice has been reinforced by the expense of current commercial devices.

To date, there has only been limited experience published regarding the usefulness of SALS for diseases of the small bowel particularly in the emergency setting. The fact that the small bowel is predominantly a mobile organ (or in the case of the terminal ileum, one that can be mobilized easily), however, makes it ideal for this approach as the focus of the operation can be controlled in its position relative to the operating instruments. This is especially the case where enterotomy or resection is required as the operating surgeon can readily exteriorize the affected segment through the single incision and perform the intended bowel procedure as in open surgery. Operative planning is also greatly helped by computerised tomography (CT) to localise and, usually, define the disease process and any locoregional effects. SALS for ileal disease therefore should allow avoidance of many of the above disadvantages.

In this cohort of consecutive, nonselected patients presenting electively and emergently for surgery over a twelve-month period, a SALS approach was used to locate and surgically manage the presenting small bowel pathology. To obviate expense (and the associated pressures of case selection) and to ensure maximum recruitment for procedural familiarity, we elected to use the “surgical glove port,” as our access device [6]. This experience is detailed herein and the advantages and considerations of this approach in this setting are discussed.

## 2. Materials and Methods

All patients presenting with ileal disease requiring surgery between October 2010 and October 2011 were considered for the SALS approach. Operations for both benign or malignant pathology of the ileum were included whether elective or urgent, and there were no exclusion criteria regarding previous surgery, body habitus, or comorbidity (once the patient was fit for laparoscopy). All patients had a CT scan of the abdomen and pelvis as the most pertinent diagnostic modality prior to surgery. Informed written consent was obtained from all patients following discussion of the potential risks and benefits of the SALS approach, and all were assured of early conversion to either a multiport or open approach in the event of this being prudent. Patient and pathology characteristics, in-hospital and 30-day postdischarge complications, length of stay, readmissions, and followup were recorded and reviewed retrospectively. Patients were contacted by telephone interview to determine the most recent outcome.

### 2.1. Preoperative Procedure

Standard perioperative management measures (including thromboembolic prophylaxis) were employed in all cases. No bowel preparation was given before surgery. Patients presenting with bowel obstruction had a nasogastric tube inserted at the time of admission.

### 2.2. Operative Procedure

**Figure 1:**
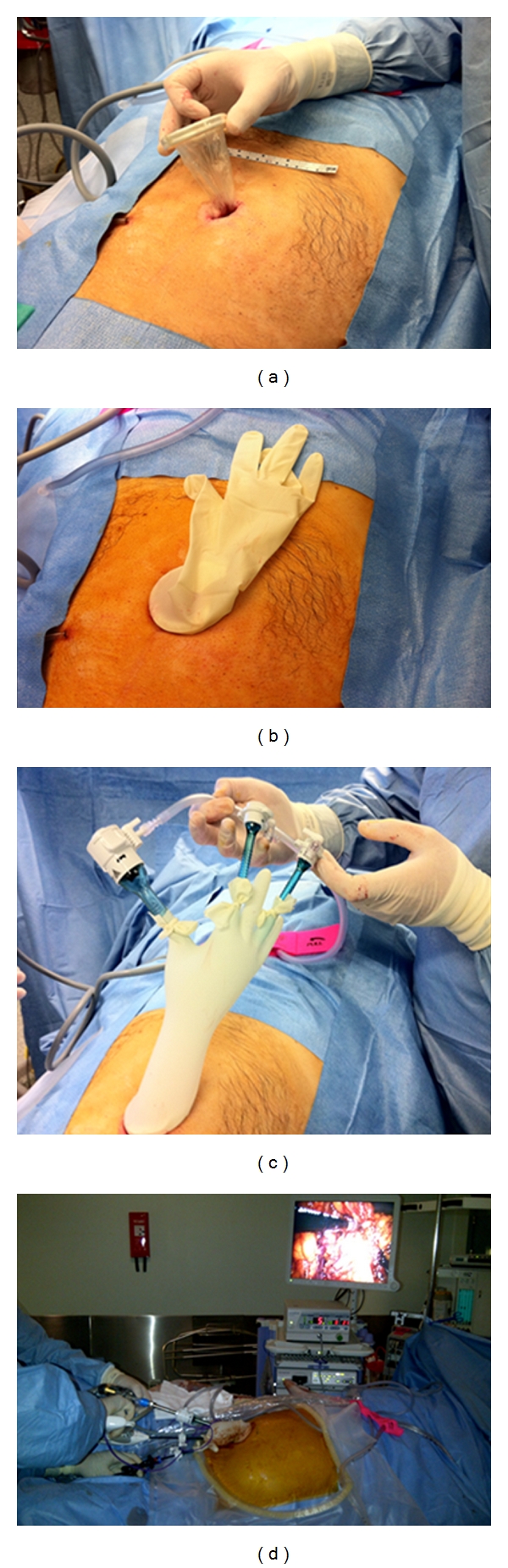
The assembly of the surgical glove port. A wound protector-retractor is placed into a 3 cm transumbilical incisions. A standard sterile surgical glove is snapped on the outer ring of the wound protector. Standard trocar sleeves are inserted into three of the fingers of the glove and secured in position by tying cut fingers from the other surgical glove in the pair around the trocars. The entire intra-abdominal component of the operation is then performed via this device as the sole abdominal access.

After the induction of general anaesthesia, prophylactic antibiotic (1.2 g co-amoxiclav in the absence of allergies) was given and the patient placed onto a bean-bag in a Trendelenburg position with both arms tucked to the side. Epidural anaesthesia was not used. After standard skin preparation (povidone-iodine) and draping, a vertical 2-3 cm skin and fascial incision centred on the patient's umbilicus was used to access the abdominal cavity. The incision was later extended if necessary to deliver the bowel and perform the resection and anastomosis. The abdominal cavity was entered carefully under direct vision. A “surgical glove port” was then constructed at the table as previously described [6]. In brief, the internal ring of a wound protector-retractor (Alexis O, Applied Medical, Rancho Santo Margarita, CA, USA) was inserted. The external ring was placed in traction and folded over itself until 2-3 cm from the abdominal surface. The surgical glove port itself was then made with one 10 mm and two 5 mm laparoscopic trocar sleeves inserted and secured in each glove finger. The glove was then stretched onto and around the outer ring which was then itself folded over again until it was in contact with the abdomen (Figure 1). The abdomen was insufflated with CO2 to a pressure of 12 mmHg. A 10 mm straight laparoscope with a 30° optic was used to visualize the abdominal cavity and standard rigid laparoscopic instrumentation used thereafter. Both surgeon and assistant stood to the patient's left side, with the camera stack to the right side. The operating table was then placed in a mild head up and right side-up position.

**Figure 2:**
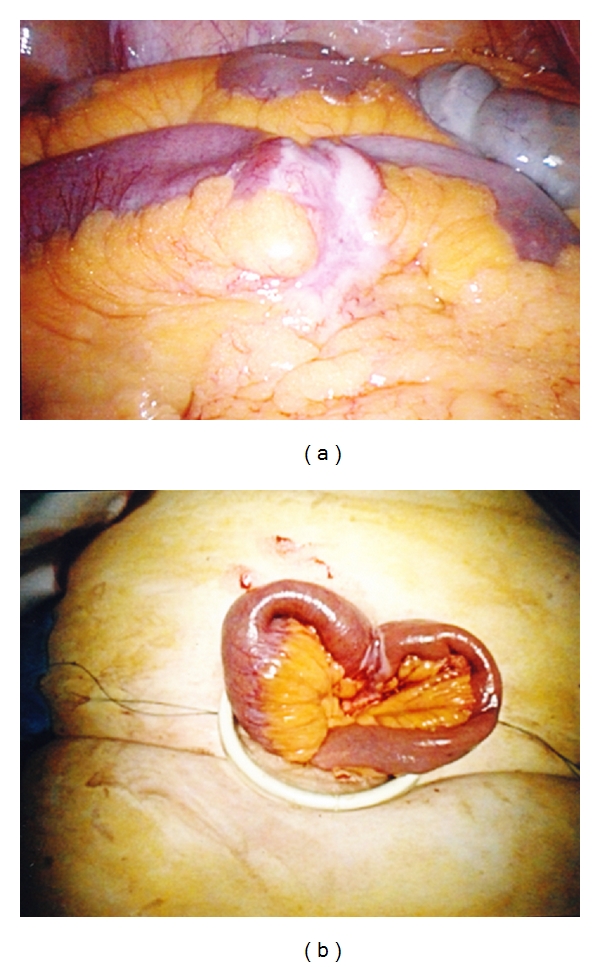
(a) Obvious small bowel pathology seen at laparoscopy (in this case, histopathological of the excised specimen proved small bowel lymphoma). (b) The same loop of small bowel as shown in Figure 2 exteriorized via the single SALS incisions to allow formal wedge excision and reanastomosis to be performed extracorporeally.

**Figure 3:**
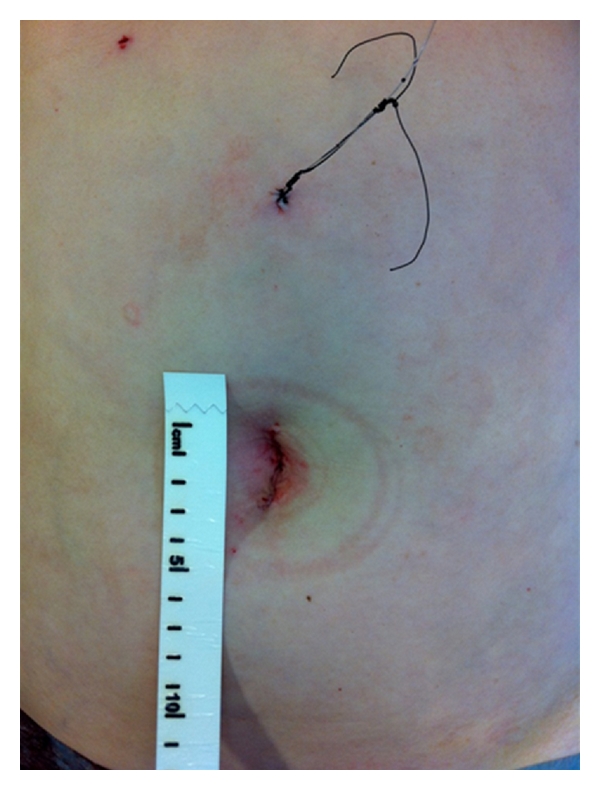
Operative photograph illustrating patient wound appearances at procedure end. The subcuticularly opposed 3 cm transumbilical wound is seen as the sole site of transabdominal access. The “Painbuster” infusional catheter is seen cephalad on the abdominal wall; this tunnelled catheter provides local anaesthesia by continual bupivacaine infusion for the first thirty hours postoperatively.

Careful inspection of abdominal cavity sometimes revealed an obvious pathology in the small bowel without further exploration (Figure 2(a)). If no pathology was seen, a thorough examination was commenced at the ileocaecal junction using two nontraumatic graspers until the pathology was located. Adhesions were divided when encountered especially in cases where they would interfere with small bowel examination or extraction. When the pathological loop of small bowel was identified, its mobility was assessed. Mobilization of right colon was only performed in cases of limited right hemicolectomy and distal ileal pathology to enable exteriorization of bowel. For exteriorisation, the bowel immediately adjacent to pathology was grasped with nontraumatic graspers. The abdomen was then deflated, the glove port disassembled, and the diseased bowel segment brought out directly through the wound protector (Figure 2(b)). Mesenteric division with Ligasure (Covidien, Dublin, Ireland) and bowel resection and functional side to side anastomosis with a straight gastrointestinal anastomosis stapler (Covidien) were performed in the usual fashion. After securing haemostasis, the bowel was reintroduced into the abdominal cavity and a second laparoscopic inspection performed after remounting the Glove port. The wound protector was then removed and fascial closure performed with interrupted monofilament suture. Skin closure was achieved with subcuticular absorbable suture. Local analgesia was then infiltrated around the wound and most often a specific infusional catheter (Painbuster, B-Braun) placed in the wound to allow continual infiltration with bupivacaine for the first 30 hours postoperatively (Figure 3).

## 3. Results

Over a ten month period, a total of ten patients (9 female and 1 male) underwent SALS for ileal disease on either an elective or urgent basis. This represents all such patients having laparoscopic surgery for this pathology over the study interval. Nine patients presented acutely with abdominal pain and/or symptoms of bowel obstruction while one presented to the clinic with iron defiency anaemia. Four patients were known already to have Crohn's disease and so were on immunosuppressive therapy. The median age of the patients was 42.5 years (range 22–78) and the median BMI was 22 kg/m^2^ (range 20.2–28). The median length of hospital stay was 4.5 days (range 2–7 days). Seven had ileal resection while two had enterotomies fashioned (one for an ileostomy and the other an ileostomy for extraction of gallstone causing ileus) and one had a mesenteric biopsy alone. Procedures included limited ileo-caecal resection (*n* = 4), ileal resection (*n* = 3), adhesiolysis (*n* = 1), enterotomy (*n* = 1), loop ileostomy (*n* = 1) and true cut biopsy (*n* = 1). Overall the mean incision length was 2.5 ± 1.0 cm (range 2.0–5.0). No patient required access modification or conversion. No intraoperative or postoperative complications were encountered. All patients tolerated normal diet within 2 days. All individual patients characteristics, presentation and perioperative data are summarized in Table 1 while their case summaries are presented next.

### 3.1. Case Summaries

**Case 1**

A 62-year-old woman (BMI 23 kg/m^2^) with a past history of hysterectomy and bilateral salpingo-oophorectomy in addition to pelvic radiotherapy for ovarian cancer presented with mid-ileal obstruction. CT abdomen demonstrated considerable distension of the proximal ileum with a clear transition point at the point of a radiopaque intraluminal focus. She underwent single-port laparoscopy which allowed adhesiolysis of considerable interloop adhesions before the obstructed loop could be determined. The obstruction was due to an intraluminal gallstone, held up in a mid-ileal loop caught by adhesions against the anterior abdominal wall. With further distal adhesiolysis, this loop was delivered up through the single-port access site allowing enterotomy, removal of the gallstone, and primary ileal closure. The patient made an uneventful recovery and was discharged home on the fifth postoperative day.

**Case 2**

A 59-year-old woman (BMI 23.5 kg/m^2^) presented with fatigue and intermittent abdominal pain in addition to iron deficiency anaemia (haemoglobin 7.5 g/dL). As both upper and lower gastrointestinal endoscopy (including terminal ileal intubation) were normal, a CT of abdomen was performed and revealed a tight distal ileal stricture with appearances consistent with either Crohn's disease or possible lymphoma. After complete mobilisation of the right colon and distal ileum, the diseased loop of bowel was exteriorised and resected. Subsequent pathological examination confirmed the diagnosis of Crohn's disease.

**Case 3**

A 78-year-old woman (BMI 25.2 kg/m^2^) presented with subacute small bowel obstruction on a background of intermittent, recurrent episodes of abdominal pain with vomiting over the previous three months. She had had no previous abdominal surgery or abdominal wall herniae on physical examination. A CT scan of her abdomen showed dilated proximal ileum with a transition point at the level of the mid-ileum but no obvious mass. Single-port laparoscopy revealed an obstructing lesion around the circumference of the bowel with mesenteric extension at this location (see Figure 2). Surgical relief was achieved by its mobilization, exteriorisation, resection, and extracorporeal anastomosis. Subsequent histological examination revealed a B-cell lymphoma.

**Case 4**

A 48-year-old woman (BMI 28 kg/m^2^) presented with a five-day history of right iliac fossa pain and tenderness. CT abdomen suggested an inflammatory focus related to her distal ileum. Single-port laparoscopy identified a cicatrising mesenteric lesion nearer to the base of her mesentery and allowed its biopsy by means of a tru-cut needle passed through a separate 2 mm stab incision. This biopsy revealed a diagnosis of a carcinoid tumor and allowed planning for its definitive resection at a subsequent operation.

**Case 5**

A 70-year-old woman (BMI 22 kg/m^2^) presented with metastatic sigmoid cancer. Due to extensive liver and lung deposits, she was treated with palliative chemotherapy without resection of the primary tumour. During her treatment, she developed signs and symptoms (pneumaturia, fecaluria, and recurrent urinary tract infections) of a colovesical fistula. To alleviate this problem, she underwent a single-port laparoscopy via a right rectus sheath incision which allowed assessment of the peritoneum and sigmoid. As the primary was unresectable, she had a defunctioning loop ileostomy fashioned in the site of the single laparoscopic access site. She was discharged home well on the second postoperative day and was able to continue her chemotherapy two weeks later.

**Case 6**

A 22 year old man (BMI 20.2 kg/m^2^) from the Middle East who presented with a three month history of recurrent abdominal pain and weight loss with night sweats having being diagnosed with pulmonary tuberculosis six months prior to presentation. CT and terminal ileoscopy revealed an inflammatory stricture of the terminal ileum. Due to the degree of local symptoms, he went single port laparoscopic resection of the ileal loop with primary stapled extracorporeal anastomosis. Histological examination demonstrated ileocaecal tuberculosis and he was commenced on appropriate therapy.

*Cases 7, 8, 9 and 10. *All females (37 years (BMI 20.8 kg/m^2^), 34 years (BMI kg/m^2^), 27 years (BMI kg/m^2^), 24 years (BMI 20.5 kg/m^2^) with known Crohn's disease presented with increasingly frequent episodes of intermittent, crampy right iliac fossa pain with occasional postprandial vomiting despite maximal medical therapy. One patient had a palpable mass evident on palpation in her right iliac fossa. CT abdomen revealed distal ileal disease in all cases. Single port laparoscopy allowed the performance of a limited ileo-caecal resection with extracorporeal anastomosis in each case. All made uncomplicated postoperative recoveries and were discharged home on between postoperative day 4 (*n* = 3) and 6. Subsequent pathological examination confirmed the diagnosis of Crohn's disease.

## 4. Discussion

SALS provides the benefits of conventional laparoscopy while reducing the tissue trauma due to the reduction in size and number of ports used. The potential benefits of SALS include reduced postoperative pain, a shorter recovery period, lower morbidity, reduced cost, and superior cosmesis [1]. It also obviates trocar-related intra-abdominal injury and port site incisional hernia formation, and thus may ultimately prove superior. This approach is particularly compelling in cases where a 3 cm incision is required anyway for the purposes of specimen extraction or stoma formation and so this wound can be made at the commencement of the surgery and used as the sole site of transabdominal incision before being closed securely under direct vision at procedure end. The ability to focus local anaesthetic regimens towards one single wound is also intuitively advantageous over the more variable responses associated with broader regional techniques such as transversus abdominus preperitoneal plane (TAPPS) blocks.

To date, however, the published experience is limited with regard to followup beyond hospital discharge and lack of long-term clinical outcome data demonstrating superiority. Furthermore, many laparoscopic surgeons still raise concerns overthe ergonomics of the technique. This is because most believe that triangulation is necessary to create the traction and counter traction that permits efficient surgery by facilitating both dissection along normal anatomical planes and laparoscopic suturing. That is why great care is taken during multiport laparoscopic surgery to respect this physical principle by ensuring trocar placement permits ideal instrument axial alignment. In contrast, the principle of triangulation hardly exists in SALS making it somewhat challenging for the laparoscopic surgeon to achieve fluent two-handed choreography for instrument movement. Therefore, there has been great interest in modification of laparoscopic instruments by implementing angulated shafts, tip reticulation, and robotic platforms to compensate for the limits of constrained parallel access [7]. At present, therefore many surgeons perhaps consider SALS best as a needlessly expensive, difficult, and time-consuming variant of minimal access surgery.

In this pilot series, we have presented a cohort of consecutive, unselected patients requiring surgery for ileal disease where a SALS access device and technique was adopted that minimizes these disadvantages while preserving the advantages of the approach. The “surgical glove port” provides more flexibility and allows greater manoeuvrability than most of the commercially available ports. The proximity of instruments within the access device, which hinders ergonomics, tends to be less constraining as the glove can stretch to increase or decrease the distance between instruments allowing greater horizontal, vertical, and rotational freedom as well as facilitate enhanced abduction and adduction of instrument tips. Furthermore, the flush positioning of the ring construct minimises the fulcrum bulk around which the instruments pivot in contrast to the majority of commercially available single-port devices which enforce parallel positioning of instrument shafts at least throughout the cylindrical component of the device. The glove port device is always readily available, thereby relieving the pressure of both preoperative selection and economic considerations and therefore means the modality can be employed with sufficient spontaneity and regularity (including its use during multiport laparoscopic colorectal resections such as to recapture the specimen extraction site to restore pneumoperitoneum and maintain full-port capacity) to ensure pan-departmental expertise [6]. Additionally a coaxial light cable instead of the tangential light cable on the laparoscope helps to overcome instrument clashing. For the novice SALS surgeon, utilizing this approach for ileal disease represents an ideal opportunity to ascend their learning curve. It is always possible to convert a SALS procedure standard laparoscopy by adding more trocars to complete the procedure (still using the single incision to extract the specimen at the end of the operation) or to extend the existing incision to convert to an open approach at no disadvantage to the patient and without significant added cost for the healthcare provider. An additional economic advantage is that, as only trocar sleeves are used with the Glove port, there is a cost-saving compared to the standard multiport approach which needs trocars with bladed obturators.

Laparoscopy is now considered an acceptable approach for initial assessment and possible management of small bowel obstruction with a conversion to a midline laparotomy rate of 29% [8]. Meta-analysis comparing laparoscopic and open approaches for the management of small bowel Crohn's disease has also demonstrated that laparoscopic surgery is associated with reduced wound infection, reduced length of stay, shorter time for recovery of enteric function, reduced reoperation rates for nondisease-related complications, and no difference in disease recurrence [9, 10]. Since the first report of SALS for the management of ileocolic Crohn's disease [11], there has been a further of four case reports [12–15] and seven case series with the number of patients ranging from one to fourteen [2, 16–21] demonstrating this approach is safe, feasible, and maintains all the advantages of traditional multiport approaches. The data presented herein further supports SALS for the management of small bowel Crohn's disease. Given the predominantly young age of patients presenting for surgery with Crohn's disease and their concerns regarding cosmesis [22] as well their potential for needing further surgery (and so the preservation of uninjured abdominal wall should facilitate reoperation), SALS may represent the optimal minimally invasive approach in this setting.

Finally, to the authors' knowledge, the usefulness and safety of this technique in the acute setting has been demonstrated for the first time. Patients presenting for urgent gastrointestinal operation have higher rates of infectious and other postoperative morbidity and greater wound complications both in the short and intermediate term [23]. If there is to be a category of patients in whom reducing the abdominal wound is important for reasons other than cosmesis, it is clearly this group of patients.

In conclusion, SALS for small bowel diseases is feasible and it can be performed without specialized instrumentation and at no extra cost. Further evaluation is required to optimise the technique; however, there are currently many available innovative, adapted techniques that can spur on the evolution of minimal access surgery by interested practitioners for the benefit of patients. While caution is needed to ensure judicious selection, ileal disease is often limited in its extent and most often specifically diagnosed by a preoperative CT. Moreover, the ileum tends to be mobile and therefore positionable both in terms of intraperitoneal quadrant and extraction via the access site.

## Figures and Tables

**Table 1:** Patients characteristics, presentation and perioperative data.

Case No	Sex	Age (yrs)	BMI (kg/m^2^)	Previous Open Abdominal Surgery	Presentation	SALS Operation	Pathology	Complications	Length of Postop Stay
1	F	62	23	Hyesterectomy & BSO	Small bowel obstruction	Adhesiolyis, enterotomy	Gallstone ileus	No	5
2	F	59	23.5	No	Abdominal pain, anaemia	Ileal resection	Crohn's Disease	No	5
3	F	78	25.2	No	Abdominal pain, vomiting	Ileal resection	Lymphoma	No	7
4	F	48	28	No	RIF pain	Trucut Biopsy	Carcinoid tumor	No	3
5	F	70	22	No	Faecaluria, recurrent UTI	Loop ileostomy	Metastatic Sigmoid cancer	No	2
6	M	22	20.2	No	Abdominal pain, weight loss	Small bowel resection	Ileal TB	No	4
7	F	37	20.8	No	RIF pain	Ileocaecal resection	Crohn's Disease	No	4
8	F	34	22	No	RIF pain	Ileocaecal resection	Crohn's Disease	No	6
9	F	27	21.5	No	RIF pain, vomiting	Ileocaecal resection	Crohn's Disease	No	3
10	F	27	21.5	No	RIF Pain with masss	Ileocaecal resection	Crohn's Disease	No	6

BMI: Body Mass Index; Postop: Postoperative; F: Female; M: Male; BSO: Bilateral Salphingo-oophorectomy; Abdo: Abdominal; RIF: Right iliac fossa; UTI: Urinary Tract Infection; TB: Tuberculosis.
